# Antibiotic stewardship for nurses: Using e-learning modules to bridge the education gap

**DOI:** 10.1017/ash.2021.216

**Published:** 2022-01-17

**Authors:** Mary T. Catanzaro

**Affiliations:** Quality Initiatives Department, The Hospital and Healthsystem Association of Pennsylvania, Harrisburg, Pennsylvania

## Abstract

**Objective:**

The Centers for Disease Control and Prevention has called for an interdisciplinary approach to antibiotic stewardship implementation that includes front-line nurses. The literature to date has identified key factors preventing uptake by nurses: lack of education, poor communication among providers, and unit culture. Three e-learning modules were developed to address the nurses’ education regarding the roles nurses play in antibiotic stewardship, antibiotic resistance, allergy assessment, medication side effects and interactions, pharmacokinetics–pharmacodynamics, culture interpretation, specimen collection, and the antibiogram. A survey was used to assess whether nurses felt more prepared to participate after finishing the modules.

**Setting:**

Front-line staff nurses in acute care were assigned e-learning modules as part of their pharmacy’s introduction of an antibiotic stewardship program for nurses.

**Methods:**

Nurses viewed the modules and completed a survey designed to rank their usefulness and to assess their attitudes.

**Results:**

Overall, 81% of nurses felt that they should be part of the antibiotic stewardship team. After completing the modules, 72% felt more empowered to participate in stewardship discussions and an additional 23% requested more education. Also, 97% felt that the information they learned could be utilized in everyday work regardless of the new program. The most cited barriers to stewardship activities were lack of education (45%) and hospital and/or unit culture (13%).

**Conclusion:**

Education and culture need to be addressed to overcome the barriers to nurses’ involvement in antimicrobial stewardship. E-learning can provide a simple and effective first step to educate nurses, with minimal time investment.

The benefits of antimicrobial stewardship are well known. Stewardship activities decrease the inappropriate use of antibiotics while encouraging their judicious and appropriate use, which leads to better patient outcomes and long-term decreases in microbial resistance.^1^ Half of inpatients in US hospitals are prescribed at least 1 antibiotic, and up to one-third of these medications are unnecessary.^2^ Globally, antimicrobial-resistant organisms are responsible for >700,000 deaths each year.^3^ This staggering statistic, as well as the urgency surrounding the threat of antimicrobial-resistant organisms and the dwindling supply of effective antibiotics, have led the Centers for Disease Control and Prevention (CDC) to require hospitals to implement antimicrobial stewardship programs by 2020.^4^ Implementation of stewardship programs are integral to other countries’ national action plans as well.^3^

Antibiotic stewardship activities have typically been the bailiwick of physicians and pharmacists, but more recently they have expanded to include a multidisciplinary team including infection preventionists, information technology staff, microbiologists, and nurses. Nurses’ contributions to activities related to stewardship have been recognized by the American Nurses Association, the CDC,^1^ and the World Health Organization. Although nurses have been recognized as integral to the advancement of stewardship programs, most literature has focused on the identification of barriers^5–8^ and less on how to incorporate existing staff into the program.^3,9,10^

The literature summarizing surveys of nurses’ attitudes and beliefs has identified perceived barriers to inclusion of this group in stewardship activities.^5–8^ It has also demonstrated how nurses’ everyday work already embodies stewardship activities such as collecting cultures prior to antibiotic initiation, obtaining an adverse drug reaction history, administering and monitoring antibiotic therapy, and conducting patient and family education.^1^ Although nurses claim these areas as being within their purview, many other areas are within their scope of their work.

The literature consistently cites 2 main areas as barriers to implementation: education and culture. Culture is more difficult to change, but the inclusion of nurses in patient rounds can help.^6^ Monsees et al^9^ recently provided practical guidance to integrate bedside nursing into antimicrobial stewardship activities. They suggested that nurses be part of the team that evaluates and collaborates on the development of antibiotic stewardship job aides.^9^ Courtenay et al,^10^ and an international team of researchers who conducted a groundbreaking study of practices in 9 countries, proposed an undergraduate competency framework to address the lack of specific education around stewardship for nurses and other health professionals that could also be adapted for practicing professionals.^10^

Education can be addressed formally in classes, presentations, webinars, and e-learning modules or informally during patient rounds or bedside discussions. Given a wide variety of clinical situations and personnel available to teach, I developed 3 e-learning modules and sought to determine whether they would be useful as a first step in implementing antibiotic stewardship for practicing nursing staff.

## Method

### E-learning modules

A review of the literature suggested topics that could enhance the bedside nurse’s understanding of antibiotic stewardship. Three modules were created using the Rise Articulate platform that covered topics identified. Themes were presented, and a short quiz tested knowledge acquisition after each module. The first module reviewed antibiotic overuse and the development of resistance, the nurses’ role in stewardship, including good infection prevention practices, and suggestions for improving communication with managers and physicians. The second module focused on microbiology–laboratory themes: appropriate collection or transport of clinical specimens, interpretation of laboratory results, and the antibiogram. The laboratory module discussed colonization versus infection, interpretation of gram stains, break points, and minimal inhibitory concentrations (MICs) for antibiotics. The pharmacy module included the CDC Core Elements of a Successful Stewardship Program, how antimicrobial resistance develops, allergic reaction versus side effects, how to take an allergy history, intravenous (IV) to oral medication conversion, de-escalation of antibiotics, drug interactions, incompatibilities, and pharmacokinetics–pharmacodynamics. The laboratory and pharmacy modules were reviewed by both microbiologists and pharmacists for accuracy and completeness. Even though all modules were built with the intent to encourage participation in antibiotic rounds or discussion with physicians, they also provided a fundamental understanding of topics that could prove useful regardless of experience, unit worked, or sustained interest in stewardship activities. The entire series took 75 minutes to complete (15 minutes for the first module and 30 minutes each for the remaining modules).

### Survey

An online Survey Monkey link immediately followed the final module and was used to assess the usefulness of the e-learning modules as well as the nurses’ attitudes toward participation in stewardship activities. The survey was not mandatory, but a certificate of course completion could be downloaded after it was taken. Information was collected regarding length of time the respondent had practiced nursing, level of education, participation in unit rounds, and perceived barriers to participation in stewardship activities. The survey asked participants to rank usefulness of subtopics in the microbiology and pharmacy modules. The survey also solicited suggestions for additional topics and queried which modules participants found most useful.

### Survey participants

Pharmacists who had previously participated in antibiotic stewardship training as part of an initiative funded by the Centers for Medicare and Medicaid Services were contacted for interest in these e-learning modules. Several hospitals uploaded the modules into their learning management system, and survey results were accepted for 18 months beginning January 2019. Because most of this period was prior to the pandemic, the pandemic should not have negatively affected results. Hospitals included a large academic medical center, as well as large and small urban hospitals in Pennsylvania, Georgia, and Louisiana. In total, 425 surveys were collected during that time. Several hospitals used the modules system-wide, and a few others targeted specific units, such as their critical care units, as they began rolling out their nursing education in phases. All surveys were included in the final analysis. Surveys were targeted for both the critical care and general ward front-line nursing staff only.

## Results

### Participant characteristics

Of 425 nurses who particpated, 204 (48%) reported 10 years of nursing experience and 106 (25%) had <3 years in practice. Also, 229 (54%) had a bachelor’s degree, 133 (31%) had an associate’s degree, and the remainder had a master’s degree.

### Participant (nurses) view of antibiotic stewardship participation

Of the 425 respondents, 346 (81%) stated that they should be involved in antibiotic stewardship, and another 55 (13%) replied that they are interested but lack time in the workday to be involved. Only 25 (6%) felt that it was not their responsibility or were not interested.

### Barriers to nurse participation

Regarding obstacles to participation in stewardship activities, 194 (45%) of 425 respondents cited lack of education about elements of stewardship and 57 (13%) felt that their hospital culture did not support their involvement. Also, 139 (33%) felt that they faced no obstacles, but 39 (9%) felt unable to adequately contribute to a discussion or did not feel it was their responsibility to do so.

### Patient rounds

Of the 425 respondents, 275 (54%) participated in physician and pharmacist patient rounds and 153 (36%) did not. For hospitals that conduct rounds, 305 (71%) participants were comfortable participating in patient rounds, and the remainder either did not feel comfortable or did not participate as part of their daily activities.

### Overall usefulness of e-learning modules

Of the 425 staff surveyed, 418 (97%) felt that they could apply some or all of the information in the modules to their daily work. Also, 310 (72%) felt that reviewing the modules helped to empower them to participate more in antibiotic discussions, and 99 (23%) requested additional education. Only 2% felt that they learned nothing they did not already know, and only 3% felt that they still did not know enough to participate in discussions with pharmacists or physicians.

### Ranking of information presented in the microbiology–laboratory module from greatest to least useful

Survey respondents were asked to rank the usefulness of the subtopics in the microbiology–laboratory module. Interpretation of laboratory tests and antibiogram interpretation were scored equally useful, whereas with the specimen collection or transport information was slightly less useful. Only 5% felt that the information presented was common knowledge to them.

### Ranking of information presented in the pharmacy module from greatest to least useful

After viewing the pharmacy module, respondents ranked subtopics as follows from most useful to least useful: IV-to-oral medication conversion, followed by antibiotic stewardship principles, antimicrobial resistance development, and de-escalation, which were all considered equally useful. Information on drug interactions, incompatibilities, and recognizing the difference between an allergic reaction and a side effect were considered the next most beneficial, followed by taking an effective allergy history. Finally, pharmacodynamics–pharmacokinetics was deemed least useful, although it was identified by 227 (53%) of the 425 respondents as having some benefit. Only 5% felt that information presented was common knowledge to them.

### Summary of most-cited free-text comments for additional educational topics

Additional topics that respondents indicated could be covered in the existing modules or in a fourth module included anticoagulant therapy, antibiotic use in sepsis, anesthetics, expansion of drug interactions, IV therapy, patient education, and specific antibiotic–resistant organisms.

## Discussion

Most nurses who completed the educational series and the survey felt that they should be part of the stewardship team. I expected greater resistance from nursing staff for having to complete the e-learning series with an expectation to participate in their stewardship program. Most respondents felt that the education provided a good foundation and was applicable to their everyday work. Nurses found both laboratory and pharmacy topics equally helpful. As expected, nurses cited education and culture as barriers to participation.

As cited in the literature,^5–8^ these 2 barriers (ie, education and culture) need to be addressed to facilitate nurses’ involvement in stewardship. Courtney et al,^11^ stressed that a multidisciplinary team needs to be involved in stewardship activities and that there needs to be an expectation of collaboration to accomplish the goal of reducing inappropriate antimicrobial use. Although topics have been identified that might benefit implementation, very little has been published regarding how to accomplish this education once nurses have begun their careers. Not all hospitals have ready access to subject-matter experts who have the time necessary to reach all staff on all shifts. Likewise, staffing shortages and increased workloads affect nurses’ ability to attend formal presentations.

This study had several limitations. It provided a snapshot of the nurses increased knowledge, and it did not include a follow-up to gauge future participation in their hospital’s stewardship programs.

In conclusion, this study has shown that e-learning can provide a simple way to educate nurses and lessen the education barrier. E-learning can equip nurses with relevant knowledge and can encourage participation in rounds by reinforcing their place in the stewardship program. This can help to integrate nurses into the interdisciplinary team seeking to reduce antibiotic overuse. At a minimum, knowledge acquired through e-learning can improve nurses’ everyday practice.
